# Pumice Characteristics and Their Utilization on the Synthesis of Mesoporous Minerals and on the Removal of Heavy Metals

**DOI:** 10.1155/2014/259379

**Published:** 2014-10-29

**Authors:** A. I. M. Ismail, O. I. El-Shafey, M. H. A. Amr, M. S. El-Maghraby

**Affiliations:** ^1^Geological Sciences Department, NRC, Dokki, Cairo 12622, Egypt; ^2^Physical Chemistry Department, NRC, Dokki, Cairo 12622, Egypt; ^3^Research Department, Trace Elements Lab, Chemistry Administration, 12 Ramsis Street, Cairo 11522, Egypt; ^4^Refractories, Ceramics and Building Materials Department, NRC, Dokki, Cairo 12622, Egypt

## Abstract

Wastewater treatment of some heavy metals was carried out by synthetic zeolite P_1_, which was prepared by alkaline hydrothermal treatment of the pumice. Both of the pumice raw materials and synthetic zeolite were investigated for their chemical phase composition, physical properties, and microstructure. The adsorption behavior of Na-zeolite P_1_ with respect to Co^+2^, Cu^+2^, Fe^+2^, and Cd^+2^ has been studied to be applied in the industrial wastewater treatment. Metal removal was investigated using synthetic solutions at different ions concentrations, time, and Na-P_1_ zeolite doses as well as constant temperature and pH. It is concluded that the optimum conditions for synthesis of highly active Na-P_1_ zeolite from natural pumice raw material are one molar NaOH concentration, temperature at 80°C, and one week as a crystallization time. In addition to the effect of time and zeolite dose as well as the ion concentration of the reaction efficiency for metals removals are recorded.

## 1. Introduction

Zeolite is a natural porous mineral described as hydrated aluminosilicates containing exchangeable alkali and earth alkaline cations (Na^+^, K^+^, Ca^+2^, or Mg^+2^) and could be synthesised from the aluminium silicate materials as clay minerals and pumice by alkali attack [1–3]. Zeolites have a large surface area, microporous structure, and high affinity for metal ions, providing an efficient scavenging pathway for heavy metals in toxic systems [4, 5]. Some of the attractive applications of natural zeolites are listed by Taffarel and Rubio [6] and Babel and Kurniawan [7]. Most of these research efforts are undertaken on laboratory scale and are at early stages of investigations to develop the synthesis methods. Synthetic zeolites are carried out in many industrialized countries in Europe, East Asia, and North America. Their consumption is dominated by companies manufacturing detergents and catalysts. Restrictions on the use of phosphate detergents have increased the demand for synthetic zeolites. Synthetic zeolites (molecular sieves) are the major alternate materials to natural zeolites. Na-P_1_ zeolite is one of the synthetic zeolites which are a class of highly porous materials. The unique structural features of these crystalline microporous solids that contain pores and cavities in the order of molecular dimensions (3–10 Å) are the main reason for their application in the realms of catalysis, separation, purification, ion exchange, and radioactive waste clean-up. More novel applications for Na-P_1_ zeolites are expected in electrochemistry, photochemistry, pharmaceutical, engineering, membrane science, and nanotechnology as their structures are expanded and more suitably engineered in a foreseeable future. It is well established that these materials that are equivalently called molecular sieves are mainly hydrated aluminosilicates. Zeolite Na-P_1_ contains two-dimensional pore system with two intersecting 8-ring channels [8]. A synthetic zeolite Na-P_1_ has similar pore structure to the natural zeolite phillipsite. Na-P_1_ zeolite is classified as high silica zeolites where it has Si/Al ratio equal to or greater than about 3. It is considered as a hydrated sodium aluminosilicate zeolite that could be synthesised from the naturally occurring alkaline volcano with NaOH attack of varying concentrations at different volcanic temperatures. Pumice is a natural rock of volcanic origin, formed by gases released during the solidification of acidic lava. The vesicular structure of pumice is formed during the eruption of gases contained in the molten viscous lava on cooling. In Egypt, pumice occasionally occurs in few locations, at the northern coast of the Mediterranean Sea at El-Arish, North Sinai [9]. The heavy metals contamination of water by the discharge of industrial wastewater of electroplating, metallurgy, chemical manufacturing, mining, and battery manufacturing is a worldwide environmental problem. Adsorption process provides an attractive alternative treatment to other removal techniques because it is more economical and readily available [10–12].

A good number of adsorbents have been used for Cd(II), Fe(II), Cu(II), and Co(II) removal from water. The adsorption of Cd(II) on red mud fitted Langmuir isotherm model having a maximum adsorption capacity of 10.57 mg g^−1^ [13]. Wang [14] used maize cobs for adsorption of Fe(III) from aqueous solution with the Langmuir adsorption capacity and the Freundlich adsorption capacity being 2.53 mg g^−1^ and 0.104 L g^−1^, respectively. The maximum adsorption capacity for Cu(II) on red mud was observed as 19.72 mg g^−1^ [13]. Gupta et al. [15] used* Spirogyra* species for removal of Cu(II) with the maximum adsorption capacity of 133.3 mg g^−1^. Use of adsorbents like Mg pellets has also been reported for Co(II) removal from water [16] and the adsorption capacity has been found to be 15.8 mg g^−1^.

The aim of this work is to study the best condition of temperature, sodium hydroxide concentration, and time required for synthesis of Na-P_1_ zeolite from the local pumice raw material and utilize the synthetic zeolite as an adsorbent for removing (Cd^+2^, Fe^+2^, Cu^+2^, and Co^+2^) ions from synthetic solutions in a batch laboratory system. Also, the effect of experimental conditions such as contact time and initial zeolite dose was described.

## 2. Materials and Methods

### 2.1. Materials

The main material used in this work is pumice grains received from El-Nile Mining Company from the Mediterranean Sea coast at El-Arish province, North Sinai, Egypt. In addition, commercial NaOH is used for alkaline hydrothermal attack for synthesis of zeolite from pumice. Hydrated chloride salts of Cu, Co, Fe, and Cd of analytical grade (AR) supplied by Merck as analytical-grade reagents and deionized water are used.

### 2.2. Methods

#### 2.2.1. Adsorbates

All adsorption experiments were carried out using an aqueous solution of CuCl_2_·2H_2_O, CoCl_2_·6H_2_O, FeCl_2_, and CdCl_2_·H_2_O. The stock solution was prepared by dissolving accurately weighted salt in distilled water to the concentration of 500 mg/L. The initial concentrations of solution were done by diluting the stock solutions in appropriate proportions to different initial concentrations.

#### 2.2.2. Adsorbents

Na-P_1_ has been prepared from pumice, which is naturally occurring alkaline volcanic rock with NaOH attack. A series of experiments were conducted to synthesis Na-P1 zeolite from locally produced pumice raw material at temperatures (80 to 120°C), NaOH solution concentrations (1–3 molar), and crystallization periods (12 h, one week, and up to one month. The dry pumice grains were well ground and passed through 63 *μ*m sieve. Pumice powder was mixed with NaOH solution in a well-sealed Teflon cylindrical vessel, heated, and stirred for 12 hours at 80°C, and then the vessel was kept in the dryer at 80–90°C for one week. The produced gel was filtered and washed well till becoming free from sodium hydroxide; the wet powder was dried for 24 h in the dryer at 110°C. The optimum conditions required for synthesis of Na-P_1_ zeolite from locally produced pumice raw material are one molar NaOH concentration, temperature at 80°C, and one week as a crystallization time. Several investigations for the adsorption of the heavy metal were carried out, including effect of contact zeolite dose, effect of time, and effect of metals concentration.

### 2.3. Characteristics of Adsorbents

The physical and chemical characteristics of prepared adsorbents such as slurry pH, point of zero charge (pH_PZC_), bulk density, and moisture were performed to determine the capability of prepared adsorbents in removing the metals Co^+2^, Cu^+2^, Fe^+2^, and Cd^+2^ from an aqueous media. For pH determination, 0.2 g of the dry, grinded adsorbent was mixed with 25 mL of distilled water and allowed to boil for 30 min in stoppered glass bottle. After cooling, the pH of the adsorbent was measured using a digital pH meter (HANNA, model pH20), allowing 5 min for the pH probe to equilibrate. Also, the point of zero charge (PZC) for the samples was determined by the following procedure: 200 mL of deionized water was added to an Erlenmeyer flask, which was then capped with stoppered glass. The deionized water was heated until boiling for 20 min to eliminate the CO_2_ dissolved in the water. The CO_2_-free water was cooled down as soon as possible and the flask was immediately capped. On the other hand, 0.5 g of each sample was weighed and placed in a 25 mL Erlenmeyer flask to which 20 mL of CO_2_-free water was added. The flask was sealed with a rubber stopper and left in continuous agitation for 72 h at 25°C. Then the solution pH was measured and this value is the point of zero charge. The bulk density was estimated by a standard procedure by weighing a known volume of gently tapped adsorbent granules. The apparent density was calculated from the volume of the graduated cylinder closely packed with the powdered sample and from the sample weight in triplicate. For moisture measurement, about 0.5 g of powdered air-dried adsorbent sample is weighed and taken in a crucible. The crucible is placed inside an electric hot-air oven and heated at 150°C for 3 hours. It is then taken out, cooled in a desiccator, and weighed. From this, the percentage of moisture can be calculated as follows: percentage of moisture = (loss in weight of adsorbent/weight of air-dried adsorbent taken) × 100.

Surface characteristics of prepared zeolite sample were characterized by scanning electron microscope (SEM) using SEM model Philips XL 30 attached with EDX unit, with accelerating voltage 30 K.V., magnification 10x up to 400.000x, and resolution for W. (3.5 nm). Samples are coated with gold. X-ray powder diffractograms, XRD, of the samples were recorded using a Bruker diffractometer (Bruker D8 advance target) and the scan rate was fixed at 8° in 2*θ* min^−1^ for phase identification. The patterns were run with Cu K*α* and secondly with monochromator (*λ* = 0.1545 nm) at 40 kV and 40 mA. Mineralogical and chemical study of the pumice were done by using X-ray fluorescence spectrometer (XRF) to identify the chemical composition. XRF, a spectrometer with wavelength dispersion PW 2400 Philips, and centrifuge Hermle were used. XRF shows that quartz, cristobalite, and albite are the main constituents in the pumice (Figure 2). SiO_2_, Al_2_O_3_, and Na_2_O are the main oxides in the pumice under investigation (93.69%) confirming the mineralogical constituents, whereas TiO_2_, MnO, MgO, Fe_2_O_3_, and CaO analyzed in the pumice under are calculated as 3.88% (Table 1). The concentrations of heavy metals in all samples were determined according to APHA using Atomic Absorption Spectrometer Unicam model 939 with graphite furnace accessory, equipped with deuterium arc background corrector. Precision of the metal measurement was determined by analyzing (in triplicate) the metal concentration of all samples and for each series of measurements an absorption calibration curve was constructed, composed of a blank and three or more standards. Accuracy and precision of the metals measurement were confirmed using external reference standards from Merck.

### 2.4. Adsorption Isotherms

The adsorption isotherm shows the equilibrium relationship of concentration in the adsorbate-adsorbent system at constant temperature.

The equilibrium adsorption isotherm is fundamentally important in the design of adsorption systems. Such adsorption isotherms may be used for scaling up batch type processes with moderate success. The shape of the equilibrium adsorption isotherm provides us with information about the adsorbent surface whether it is homogeneous or heterogeneous. Also, its study is helpful in evaluating the maximum adsorption capacity of adsorbate for the given adsorbent. For adsorption isotherms models were used in this work, Langmuir and Freundlich isotherms. They differ in their assumption, shape of the isotherm, and nature of the adsorbent surface.

### 2.5. Langmuir Isotherm Model

Langmuir (1918) model assumes that uptake of metal ion occurs on a homogeneous surface by monolayer adsorption without any interaction between adsorbed ions. The model is also based on the assumption that all the sorption sites are energetically identical and sorption occurs on a structurally homogeneous sorbent. The linear form of Langmuir is given as follows:

(1) $$\begin{array}{c} \frac{C_{e}}{q_{e}}=\frac{1}{K_{L}Q_{m}}+\frac{1}{Q_{m}}C_{e}. \end{array}$$

Thus, a linear plot of *C*_*e*_/*q*_*e*_ versus *C*_*e*_ confirms the validity of the Langmuir giving correlation coefficients (*R* ^2^) close to unity, and *K*_*L*_ is a constant related to the free energy of adsorption as expressed (*K*_*L*_ *αe* ^−ΔG/RT^). Deviations from the basic assumption of the Langmuir model do limit interpretation of the values for *Q*_*m*_ and *K*_*L*_ in terms of absolute surface areas and sorption free energies. The free energy of adsorption, Δ*G*, can also be evaluated from the parameter *K*_*L*_ according to the expression Δ*G* = RTln⁡⁡*K*_*L*_. Values of *Q*_*m*_ and *K*_*L*_ are obtained from slope and intercept of *C*_*e*_/*q*_*e*_ versus *C*_*e*_ plot, respectively. A further analysis of the Langmuir equation can be made on the basis of a dimensionless equilibrium parameter, *R*_*L*_ (known as the separation factor which is considered as a more reliable indicator of adsorptions). This parameter (*R*_*L*_) can be expressed by Weber and Chakkravorti (1974):

(2) $$\begin{array}{c} R_{L}=\frac{1}{1+bC_{i}}, \end{array}$$

where *C*_*i*_ is the initial concentration metal ion (mg/L) and *b* (L mg^−1^) is the Langmuir constant described above.

Also, the influence of the isotherm shape on whether the adsorption is “favorable” or “unfavorable” at highest initial concentration of the dye, *C*_*o*_, can be described by a term “*R*_*L*_”, a dimensionless constant separation factor in equation; calculated value for *R*_*L*_ indicates the nature of adsorption process as given: irreversible (*R*_*L*_ = 0), favorable (0 < *R*_*L*_ < 1), linear (*R*_*L*_ = 1), and favorable (*R*_*L*_ > 1).

### 2.6. Freundlich Isotherm Model

Freundlich isotherm is an empirical equation that can be described as the reversible adsorption onto heterogeneous surface at sites with different energy of adsorption and is not restricted to the formation of the monolayer of adsorbate. The nonlinear form of this model is expressed as

(3) $$\begin{array}{c} q_{e}=K_{F}{C_{e}}^{1/n}, \end{array}$$

where *K*_*F*_ is the Freundlich constant (mg/g)/(L/mg)1/*n* and also referred to as adsorption capacity, while *n* is the heterogeneity factor and related to adsorption intensity. The parameter *n* gives an indication of the adsorption type whether is linear (*n* = 1) or a physical process (*n* > 1) is favorable, or a chemical process (*n* < 1). On the other hand, the value of 1/*n* < 1 or 1/*n* > 1 indicates a normal Langmuir isotherm and cooperative adsorption, respectively. Freundlich model can be represented by linear form as follows:

(4) $$\begin{array}{c} \ln q_{e}=\ln K_{F}+\frac{1}{n}\ln C_{e}. \end{array}$$

A plot of ln⁡*q*_*e*_ versus ln⁡*C*_*e*_ gives a straight line, where the values of *K*_*F*_ and 1/*n* are determined from the intercept and the slope, respectively.

### 2.7. Adsorption Kinetic Experiments

The kinetic tests were carried out following the same procedure used for the equilibrium tests. Aqueous samples were taken at different intervals of time and the concentrations of adsorbent were measured at the same intervals. The amount of absorbed onto the developed adsorbents at time *t* (min), *q*_*t*_ (mg/g) was calculated by means of the expression below:

(5) $$\begin{array}{c} q_{e}=\frac{\left(C_{0}-C_{t}\right)V}{m}, \end{array}$$

where *C*_*o*_ and *C*_*t*_ are the liquid-phase concentrations at an initial and predetermined time *t* (mg/L), respectively, *V* is the volume of solution (L), and *m* is the dry weight of the added adsorbent (g). The kinetic data were then carried out for the pseudo-first-order and pseudo-second-order.

### 2.8. Pseudo-First-Order Kinetic Model

The rate constant of adsorption was determined from the pseudo-first-order equation:

(6) $$\begin{array}{c} \log\left(q_{e}-q_{t}\right)=\log q_{e}-\frac{k_{1}t}{2.303}, \end{array}$$

where *q*_*e*_ and *q*_*t*_ (mg/g) are the amounts of adsorbed (mg/g) at equilibrium and at time *t* (min), respectively, and *k*_*t*_ is the adsorption rate constant (min⁡^−1^).

### 2.9. Pseudo-Second-Order Kinetic Model

The pseudo-second-order equation based on the equilibrium adsorption is expressed as

(7) $$\begin{array}{c} \frac{t}{q_{t}}=\frac{1}{k_{2}{q_{e}}^{2}}+\frac{t}{q_{e}}, \end{array}$$

where *k*_2_ (g/mg·min) is the rate constant of second-order adsorption.

## 3. Results and Discussions

### 3.1. Characterization of Pumice and Synthetic Zeolite

**Figure 1:**
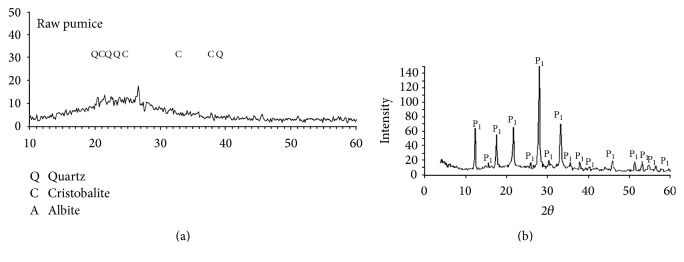
(a) XRD of the pumice pattern under study. (b) XRD of the synthesis of Na-P_1_ zeolite pattern.

**Figure 2:**
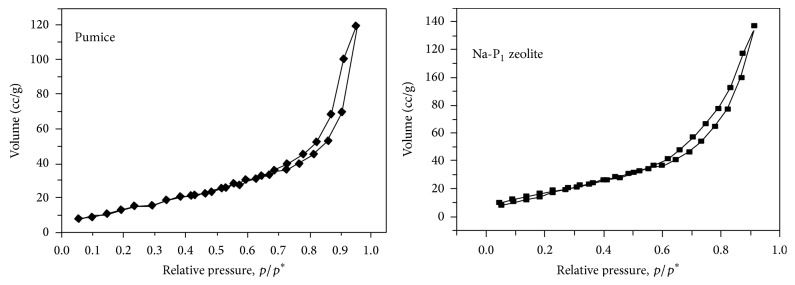
N_2_—adsorption-desorption isotherms of pumice and Na-P_1_ zeolite.

**Figure 3:**
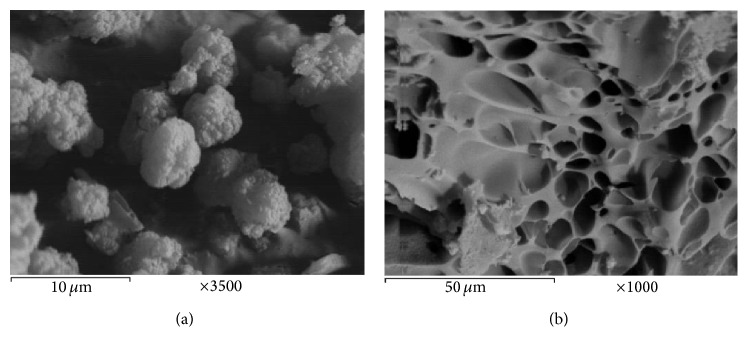
SEM photograph of (a) zeolite P_1_ and (b) pumice.

Some physical and chemical features of pumice and Na-zeolite P_1_ are listed in Table 2. It is evident that the treatment with NaOH has a significant efficiency on the physicochemical properties of synthesized adsorbent Na-zeolite P_1_. The pH of the aqueous slurry and pH_PZC_ of adsorbents may give a good indication about the surface oxygen complexes and the electronic surface charges of adsorbents. This surface charge arises from the interaction between adsorbent surface and the aqueous solution. The complexes on adsorbent surface are generally classified as acidic, basic, or neutral. Based on the slurry pH, the nature of surface oxygen groups on the support and the dominant complexes can be deduced. From Table 2, it is clear that the pH is higher than the pH_PZC_; the surface of the adsorbent is negatively charged, favoring the adsorption of cationic species (Co^+2^, Cu^+2^, Fe^+2^, and Cd^+2^). Bulk density of the pumice and Na-zeolite P_1_ decreased from 0.85 to 0.50 mg/g and the moisture is 0%. This finding is due to the generation of porous structure and the increase in binding sites after chemical modification. Chemical and phase composition as well as microstructure of the pumice raw material have been studied. The data obtained from the XRF and XRD are discussed before [17]. Table 1 illustrates that SiO_2_ is the major component (~71.0%) with about 14.20% Al_2_O_3_ as well as a total of about 12.4% fluxing oxides. XRD pattern in Figure 1 confirms the glassy phase in addition to minor crystalline quartz phase, which is the only phase. The amorphous nature of the silicate pumice as well as the high content of the pore size with variable distributions is ensured by the SEM in Figure 3(b). Phase composition and microstructure of the synthetic zeolite studied by XRD (Figure 2) and SEM reveal that the zeolite P_1_ is the only phase formed during these conditions of alkali treatment during the alkaline hydrothermal attack of locally produced pumice raw material at the optimum condition of synthesis. The micrograph of synthetic zeolite shows very fine agglomerated crystalline grains (gray) Figure 3(a). XRD analysis was used to prove the formation of Na-zeolite P_1_ before and after treatment with NaOH. The X-ray diffractogram of pumice shows that it was considered a glass because it has no crystal structure. Pumice is an unusually light rock due to the many bubbles inside it, Figure 3(b). Pumice has an average porosity of 90% and initially floats on water. Pumice varies in density according to the thickness of the solid material between the bubbles; many samples float in water. It is formed by volcanic eruptions when molten lava is shot in the air with many bubbles of gas in it. As it cools, it solidifies into pumice. The treatment with NaOH leads to the formation of Na-zeolite P_1_ which has higher degree of crystallinity, Figure 3(a). XRD analysis was used to prove the formation of Na-zeolite P_1_ before and after treatment with NaOH.

### 3.2. Adsorption Experiments

Adsorption isotherm and kinetics of Co^+2^, Cu^+2^, Fe^+2^, and Cd^+2^ on the synthetic zeolite were investigated using batch experiments. Equilibrium and kinetic studies were carried out on the removal of Co^+2^, Cu^+2^, Fe^+2^, and Cd^+2^ from aqueous solution using synthetic zeolite P_1_. Adsorption test on prepared adsorbent was studied using a batch process. For this purpose, the effects of adsorbents dose (0.1–1 g), contact time (1–8 min), and metal concentration (300–700 mg/L) on the adsorption of Co^+2^, Cu^+2^, Fe^+2^, and Cd^+2^ (25 mL of 100 mg/L) were evaluated during the present study. For all the above-mentioned equilibrium and kinetic studies, the mixture of adsorbent and solution was magnetically stirred at 150 rpm for 30 min as determined from kinetic tests.

### 3.3. Effect of Zeolite Dose

**Figure 4:**
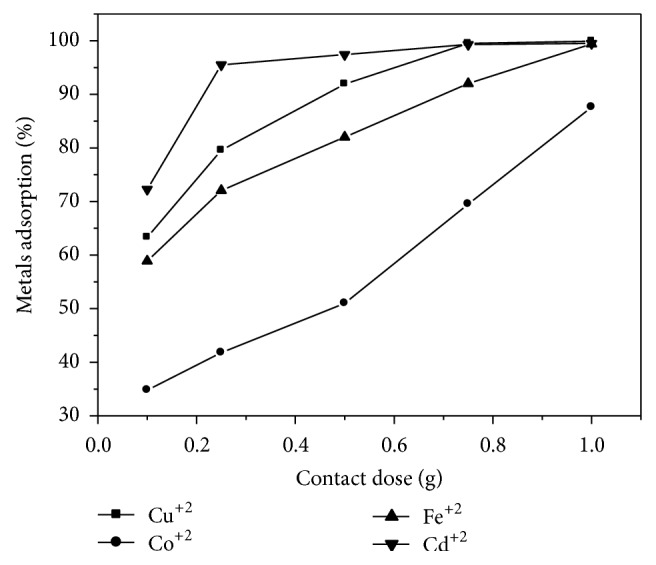
Adsorption of metals' ions by zeolite as a function of its weight.

Batch adsorption experiments were conducted by mixing 50 cm^3^ solutions of 500 ppm containing one metal of Co^+2^, Cu^+2^, Fe^+2^, and Cd^+2^ with a constant dose of Na-zeolite P_1_ (0.1 g, 0.25 g, 0.5 g, 0.75 g, or 1 g) in 100 cm^3^ Pyrex Erlenmeyer flasks with cap. Flasks were shaken (150 rpm) at 25 ± 1°C using a Brunswick Scientific G25KC incubator orbital shaker. It is observed that the percentage adsorption for Co^+2^, Cu^+2^, Fe^+2^, and Cd^+2^ ions increases with increasing zeolite weight in aqueous solutions as illustrated in Figure 4. The maximal exchange levels attained were as follows: Cd^+2^ > Fe^+2^ > Cu^+2^ > Co^+2^. The heavy metal uptake may be attributed to different mechanisms of ion-exchange processes as well as to the adsorption process [18–20]. During the ion-exchange process, metal ions had to move not only through the pores of the Na-zeolite P_1_ mass, but also through channels of the lattice, and they had to replace exchangeable cations (mainly sodium). Diffusion was faster through the pores and was retarded when the ions moved through the smaller diameter channels. In this case the metal ion uptake 6 could mainly be attributed to ion-exchange reactions in the microporous minerals of the Na-zeolite P_1_ samples.

### 3.4. Effect of Contact Time

**Figure 5:**
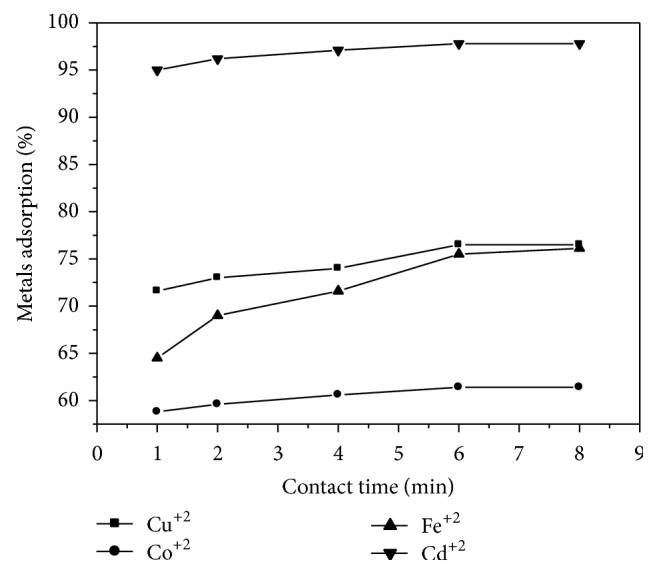
Adsorption of metals' ions by zeolite as a function of contact time.

**Figure 6:**
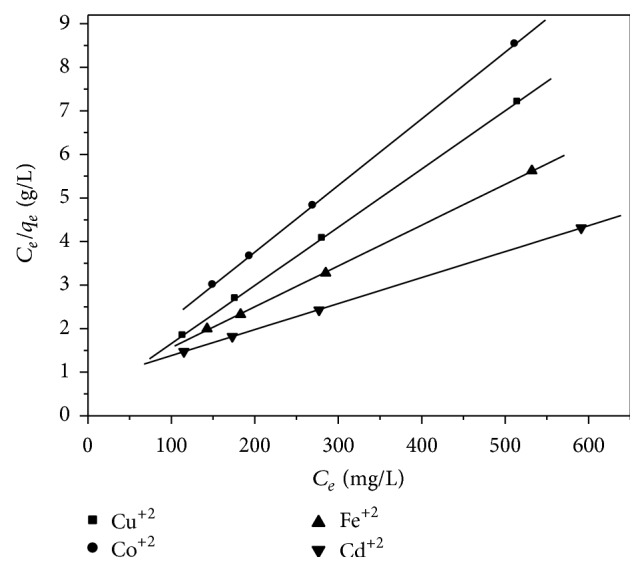
Langmuir plots for metal ions adsorption onto zeolite P_1_.

By varying the contact time (1, 2, 4, 6, and 8 min) while keeping all other parameters constant with respect to pH, temperature, and dose of Na-zeolite P_1_, 0.25 gm, the influence of contact time on the zeolite capacity for different metal ions is shown in Figure 5. Removal efficiency increases for Cadmium from 95 to 98%, for copper from 71 to 76%, for iron from 64 to 76%, and for cobalt from 58 to 61% as the time increases from 1 to 8 min.  Figure 6 represents the variation in metal ion adsorption with time; however the adsorption rate becomes slower with passage of time up to 4 min. The initial faster rate of removal of each metal ion may be due to the availability of the uncovered surface area of the adsorbents. The slight increase in the percentage of adsorption of each metal adsorbent is Cd^+2^ > Fe^+2^ > Cu^+2^ > Co^+2^. This may be attributed to the difference in nature between metal ions with respect to Na-zeolite P_1_ surface. That may be attributed to different mechanisms of ion-exchange processes as well as the adsorption process [18–20].

### 3.5. Effect of Metals Concentration

The uptake of Cd(II), Cu(II), Ni(II), Pb(II), and Zn(II) onto Na-zeolite P_1_ as a function of their concentrations was studied at 25 ± 0.1°C by varying the metal concentrations 300, 400, 500, and 700 mg/L while keeping all other parameters constant with respect to dose and time.

### 3.6. Adsorption Isotherms

For adsorption isotherms models were used in this work, Langmuir and Freundlich isotherms. They differ in their assumption, shape of the isotherm, and nature of the adsorbent surface.

### 3.7. Langmuir Isotherm Model

**Figure 7:**
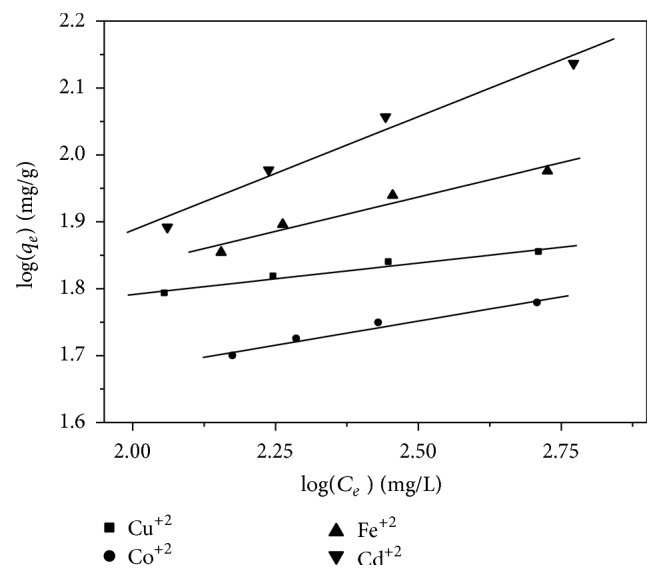
Freundlich plots for metal ions adsorption onto zeolite.

Langmuir (1918) isotherm which models the monolayer coverage of the sorbent surface assumes that sorption occurs at specific homogeneous sorption site. The model is also based on the assumption that all the sorption sites are energetically identical and sorption occurs on a structurally homogeneous sorbent [18–20].  Figure 7 shows that the Langmuir model effectively and significantly described the sorption data with all *R* ^2^ values ≥0.96.  Table 3 shows the relation between the ionic radius, electronegativity, and Langmuir constant. The maximum sorption capacity (*K*_*L*_) represents monolayer coverage of sorbent; according to the *K*_*L*_ (mg/g) parameter, monolayer capacities of zeolite are arranged in the following sequence: Cd^+2^ > Fe^+2^ > Cu^+2^ > Co^+2^. These metals seem to reach saturation, which means that the metal had clogged possible available sites in zeolite and further adsorption could take place only at new surfaces [21]. The sequence of adsorption of several heavy metals are not correlated either with the sequence of ionic radii or with the sequence of electronegativity. There is, however, a parallel relation between the adsorption sequence and the hydrolysis properties of the heavy metal cations, as pointed out by several investigators [22–24].

### 3.8. Freundlich Isotherm Model

**Figure 8:**
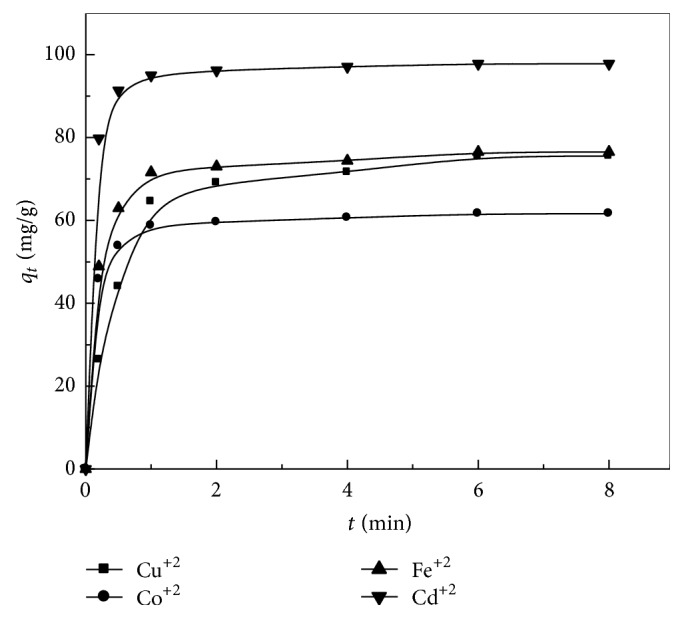
Amount of metal ions adsorbed onto zeolite versus time at 25°C.

This isotherm developed by Freundlich (1926) describes the equilibrium on heterogeneous surfaces and does not assume monolayer capacity. This isotherm gives an expression encompassing the surface heterogeneity and the exponential distribution of active sites and their energies. A linear plot is obtained when log *C*_*e*_ is plotted against log *q*_*e*_ over the entire concentration range of metal ions investigated and the values of *K*_*F*_ (mg/g) and *n* can be calculated from the intercept and the slope of this straight line, respectively (Figure 8). The arrangement of heavy metals according to dose required for the reduction of metal concentrations in Freundlich equation is Cd^+2^ > Fe^+2^ > Cu^+2^ > Co^+2^. The adsorption isotherm data were fitted well to the Langmuir model from Langmuir model equations.

### 3.9. Sorption Kinetics

**Figure 9:**
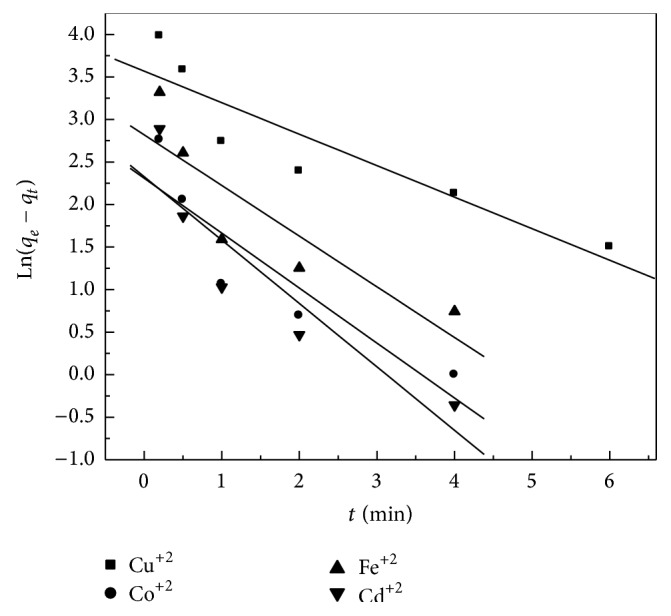
Pseudo-first-order sorption kinetics for the sorption of metal ions onto zeolite at 25°C.

**Figure 10:**
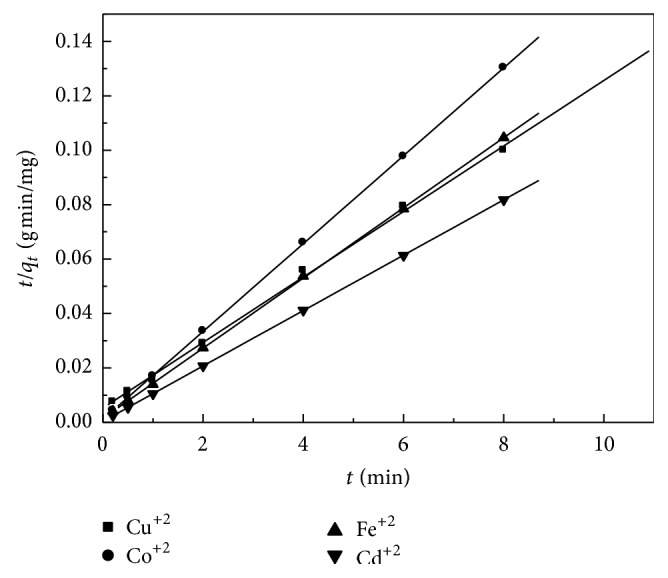
Pseudo-second-order sorption kinetics for the sorption of metal ions onto zeolite at 25°C.

In order to investigate the adsorption kinetics of heavy metals on the prepared zeolite adsorbent, three kinetic models have been tested: pseudo-first-order. The calculated parameters from pseudo-first-order and pseudo-second-order models (Figures 9 and 10) are summarized in Table 4. According to the pseudo-first-order model, the values of *k*_1_ and *q*_*e*_ were calculated from the slope and intercept from the plots of log⁡⁡ (*q*_*e*_ − *q*_*t*_) versus *t*. It was found that the experimental values were not adequate with the calculated ones (Table 4), indicating that the first-order model does not reproduce the adsorption kinetic of heavy metals on the prepared adsorbents. In case of the pseudo-second-order model, the calculated values of kinetic studies showed that the zeolite is a good potential as adsorbent for (Co^+2^, Cu^+2^, Fe^+2^, and Cd^+2^) ions follows the pseudo-second-order, and, hence, this model is more likely to predict the behavior over the whole experimental range of H M adsorption more than pseudo-first-order model. Also, the values of correlation coefficients (2) are close to unity for pseudo-second-order model rather than the pseudo-first-order one; that is, the kinetic removal of H M is quite described by pseudo-second-order model.

## 4. Conclusion

The alkaline treatment of very cheap local pumice leads to the formation of Na-zeolite P_1_ which has the high degree of crystallinity, the pH is higher than the pH_PZC_, and the surface of the adsorbent is negatively charged, favoring the adsorption of cationic species (Co^+2^, Cu^+2^, Fe^+2^, and Cd^+2^). The adsorption kinetics study of the heavy metals followed a pseudo-second-order model. This indicates that the adsorption may be controlled by chemical adsorption. Both the Langmuir and Freundlich models fit equilibrium data well implying the existence of a monolayer adsorption and a heterogeneous surface existence in Na-zeolite P_1_, showing a strong adsorption capacity for (Co^+2^, Cu^+2^, Fe^+2^, and Cd^+2^) ions reaching a maximum capacity for Cd^+2^. Heavy metal ions uptake is the result of a combination of several interfacial reactions, namely, ion exchange, chemisorption, and adsorption as potential determining ions. Results found showed that the modified zeolite shows a good potential as adsorbent for (Co^+2^, Cu^+2^, Fe^+2^, and Cd^+2^) ions.

## Figures and Tables

**Table 1:** Chemical composition of the pumice determined by X-ray fluorescence.

Oxides	wt.%
SiO_2_	70.97
TiO_2_	00.14
Al_2_O_3_	14.24
MnO	0.140
MgO	0.350
Fe_2_O_3_	1.880
CaO	1.370
Na_2_O	4.460
K_2_O	4.020
LOI	2.410

**Table 2:** Physicochemical characteristics of the pumice and zeolite P_1_.

Characteristics	Pumice	Zeolite P_1_
Slurry pH	6.0	9.0
pH_PZC_	5.0	8.0
Bulk density, g/cm^3^	0.85	0.5
Moisture, %	1.0	1.0
*S*_BET_, m^2^/g	31	40
*V*_*P*_, cm^3^/g	0.01	0.02
*r*_*p*_, Å	2.0	2.2

**Table 3:** Characteristic parameters and correlation coefficients of the experimental data according to Langmuir and Freundlich equations on zeolite at 25 ± 0.1°C.

Metal	Ionic radius^*^ (Å)	Electro negativity^**^	Langmuir constant			Freundlich constant		
			*K*_*L*_ (mg/g)	*b* (L/mg)	Correlation coefficient (*R* ^2^)	*K*_*F*_ (mg/g)	*n*	Correlation coefficient (*R* ^2^)
Co^+2^	1.88	1.88	1.27	23.99	0.986	16.16	0.340	0.983
Cu^+2^	1.30	1.90	1.59	45.70	0.986	24.60	0.144	0.999
Fe^+2^	1.83	1.83	2.86	67.12	0.986	26.50	0.205	0.985
Cd^+2^	1.69	1.69	3.12	131.9	0.999	40.00	0.094	0.976

^*^Ionic radius (Å) and ^**^electronegativity from [25].

**Table 4:** Kinetic parameters of sorption for the metal ions onto zeolite at 25°C.

Metal	Pseudo-first-order				Pseudo-second-order		
	*q*_*e* exp⁡_ (mg/g)	*q*_*e* cal_ (mg/g)	*K*_1_ (min^−1^)	*R*_1_^2^	*q*_*e* cal_ (mg/g)	*K*_2_ (g/mg min)	*R*_2_^2^
Co^+2^	61.60	10.06	−0.3705	0.8357	61.95	0.2326	0.9999
Cu^+2^	75.50	10.24	−0.6458	0.8170	77.57	0.1123	0.9994
Fe^+2^	76.50	16.76	−0.5954	0.7651	80.05	0.0274	0.9999
Cd^+2^	97.80	35.45	−0.7452	0.8337	98.32	0.2650	1
